# Pre-endoscopy SARS-CoV-2 testing strategy during COVID-19 pandemic: *the care must go on*

**DOI:** 10.1186/s40001-022-00672-5

**Published:** 2022-03-18

**Authors:** M. Casper, M. C. Reichert, J. Rissland, S. Smola, F. Lammert, M. Krawczyk

**Affiliations:** 1 Department of Medicine II – Gastroenterology, Hepatology and Endocrinology, Saarland University Medical Center, Saarland University, Kirrberger Straße 100, 66421 Homburg, Germany; 2 Institute of Virology, Saarland University Medical Center, Saarland University, Homburg, Germany; 3 Hannover Medical School, Hannover Health Sciences Campus, Hannover, Germany; 4 Laboratory of Metabolic Liver Diseases, Department of General, Transplant and Liver Surgery, Center for Preclinical Research, Medical University of Warsaw, Warsaw, Poland

**Keywords:** Gastrointestinal endoscopy, COVID-19 diagnostic testing, Gastrointestinal diseases

## Abstract

**Background:**

In response to the COVID-19 pandemic, endoscopic societies initially recommended reduction of endoscopic procedures. In particular non-urgent endoscopies should be postponed. However, this might lead to unnecessary delay in diagnosing gastrointestinal conditions.

**Methods:**

Retrospectively we analysed the gastrointestinal endoscopies performed at the Central Endoscopy Unit of Saarland University Medical Center during seven weeks from 23 March to 10 May 2020 and present our real-world single-centre experience with an individualized rtPCR-based pre-endoscopy SARS-CoV-2 testing strategy. We also present our experience with this strategy in 2021.

**Results:**

Altogether 359 gastrointestinal endoscopies were performed in the initial period. The testing strategy enabled us to conservatively handle endoscopy programme reduction (44% reduction as compared 2019) during the first wave of the COVID-19 pandemic. The results of COVID-19 rtPCR from nasopharyngeal swabs were available in 89% of patients prior to endoscopies. Apart from six patients with known COVID-19, all other tested patients were negative. The frequencies of endoscopic therapies and clinically significant findings did not differ between patients with or without SARS-CoV-2 tests. In 2021 we were able to unrestrictedly perform all requested endoscopic procedures (> 5000 procedures) by applying the rtPCR-based pre-endoscopy SARS-CoV-2 testing strategy, regardless of next waves of COVID-19. Only two out-patients (1893 out-patient procedures) were tested positive in the year 2021.

**Conclusion:**

A structured pre-endoscopy SARS-CoV-2 testing strategy is feasible in the clinical routine of an endoscopy unit. rtPCR-based pre-endoscopy SARS-CoV-2 testing safely allowed unrestricted continuation of endoscopic procedures even in the presence of high incidence rates of COVID-19. Given the low frequency of positive tests, the absolute effect of pre-endoscopy testing on viral transmission may be low when FFP-2 masks are regularly used.

## Introduction

COVID-19 substantially affects health care systems worldwide. Gastroenterologists must balance the risk for SARS-CoV-2 virus transmission during endoscopy against the risk caused by the delay of the procedures. Endoscopic societies around the world have published recommendations to assist endoscopists in decision-making during the pandemic [1]. In most of these recommendations it is advised to use personal protective equipment (PPE) and to postpone non-urgent or routine procedures temporarily. However, urgent procedures are not uniformly defined, requiring case-by-case decisions from endoscopists in many patients. Following these recommendations, endoscopy programmes were substantially reduced worldwide (on average by 83%) during the pandemic [2]. The short- and medium-term consequences of this approach are difficult to estimate. As an alternative we present here our real-world experience with the rtPCR-based pre-endoscopy SARS-CoV-2 testing strategy that enabled us to conservatively handle endoscopy programme reduction.

## Patients and methods

### Study cohort and background

Retrospectively we analysed the gastrointestinal endoscopies performed at the Central Endoscopy Unit of Saarland University Medical Center during seven weeks from 23 March to 10 May 2020 (local peak period during the first wave of the COVID-19 pandemic). This endoscopy unit is an academic tertiary referral centre serving all medical and surgical departments as well as state and regional health care providers. In Germany, more than 169,000 COVID-19 cases leading to more than 7,400 deaths (4.4%) had been reported until 10 May 2020. The current cumulative incidence in Saarland, which is one of the Federal states in Southwestern Germany at the border to France, is 279 cases per 100,000. Between 23 March and 10 May 2020, 2,514 individuals were tested positive for SARS-CoV-2 in Saarland, of whom 146 (5.8%) died.

### Testing strategy

The rtPCR-based pre-endoscopy SARS-CoV-2 testing on nasopharyngeal swabs (Roche, Basel, Switzerland Altona Diagnostics, Hamburg, Germany) was broadly offered to in-patients in the Saarland University Medical Centre during the pandemic. Starting on 2 April 2020, structured rtPCR testing was also implemented for all out-patients scheduled for endoscopic procedures. Endoscopies were planned to be performed when the patient tested negative within 5 days prior to the procedure. In addition, all members of the endoscopy suite (nurses, physicians, endoscope reprocessing and cleaning staff) were tested weekly using pooling of samples, as described recently [3].

Routine rtPCR test results were available within 3–5 h, when performed before 04:30 PM on weekdays or 02:00 PM on weekends, or on the next day when performed later. Emergency testing was available 24/7 within 3 h. Patients were also inquired using questionnaires for typical COVID-19 symptoms prior to endoscopy. All necessary in-patient endoscopic procedures were performed as requested (i.e. endoscopic procedures to evaluate clinically significant gastrointestinal symptoms leading to hospital admission or prolonging/complicating hospital stay: gastrointestinal bleeding, symptomatic pancreaticobiliary disease, suspected gastrointestinal malignancy, IBD flare, therapeutic endoscopy for time-sensitive diagnoses). For out-patients, urgent procedures were unrestrictedly offered (i.e. suspected upper or lower gastrointestinal bleeding, suspected malignancy, dysphagia, patients with time-sensitive diagnosis, IBD patients when endoscopy may change management). Elective procedures were discussed on an individual basis with patients and referring physicians: Generally, endoscopy was offered to all symptomatic patients (abdominal pain, diarrhoea), whereas delay of endoscopy was recommended for asymptomatic patients scheduled for screening or surveillance endoscopies (colorectal cancer screening, oesophageal varices or Barrett’s oesophagus surveillance, follow-up for gastric ulcers without suspicion of malignancy). When patients insisted on performing endoscopy, this was done also in asymptomatic patients scheduled for elective procedures.

Risk-stratified personal protective measures conformed with the published recommendations [4]. Asymptomatic patients with a negative rtPCR test result were categorized as low-risk patients and PPE was adapted (surgical mask, goggles, single-use gown, gloves, hairnet).

### Strategy in the period after the first wave and during following waves of COVID-19 pandemic

After the first wave of COVID-19 we continued and optimized the testing strategy developed during the first wave: All in-patients had a rtPCR test within 24 h prior to or at admission, so that a negative test was available at the moment of endoscopy. With the availability of antigen-detecting rapid diagnostic tests, such a test was always available at the time of emergency endoscopic procedures. In all out-patients scheduled for endoscopy a rtPCR test was performed at our unit 48 h prior to the endoscopy together with informed consent. We tested all patients irrespective of vaccination status, prior COVID-19 infection or type of endoscopic procedure. With this strategy all endoscopic procedures requested were carried out without restrictions irrespective of indication throughout the year 2021. This also included the second (week 40/2020–week 8/2021; Saarland peak 7-day incidence rate 202 on 10 January), third (week 9/2021–week 23/2021; Saarland peak 7-day incidence rate 149 on 27 April) and fourth wave of the pandemic in Germany (ongoing until week 41/2021; Saarland peak 7-day incidence rate 440.9 on 29 November).

## Results

### The first wave (period 23 March to 10 May 2020)

During the analysed period, we performed 359 gastrointestinal endoscopic procedures. In the same period in 2019 a total of 626 (42.7% reduction) endoscopies were done (187, including 10 PEGs vs. 340 upper endoscopies [55%], 121 vs. 212 lower endoscopies [57.1%], 42 vs. 58 ERCPs [72.4%]). In total, 263 (73.2%) procedures were in-patient endoscopies (2019: 423; 67.6%). Six endoscopies performed in COVID-19 patients on extracorporeal membrane oxygenation were excluded from further analysis [5]. Table 1 summarizes the indications, outcomes and baseline characteristics of 353 endoscopies included in the final analysis. A majority of endoscopies were routine endoscopies (*n* = 268), while 40 emergency endoscopies were done within 6 h and 45 within 24 h of presentation.

**Table 1:** Endoscopic procedures performed between 23 March 2020 and 10 May 2020

EGD *n* = 184; age 60 ± 17 years; 72 females (39%); 59 out-patients (32%); 160 available rtPCR SARS-CoV-2 (87%)		
Indication		Outcome [normal; endoscopic therapy; changed management]
1)Emergency/essential	50 (27%)	12; 56; 74%
2)Urgent	51 (28%)	26; 24; 44%
3)Non-urgent symptomatic	59 (32%)	31; 0; 7%
4)Non-urgent asymptomatic	24 (13%)	21; 8; 0%
Colonoscopy *n* = 118; age 64 ± 17 years; 49 females (42%); 37 out-patients (31%); 106 available rtPCR SARS-CoV-2 (90%)		
Indication		Outcome [normal; endoscopic therapy; changed management]
1)Emergency/essential:	28 (24%)	11; 36; 75%
2)Urgent:	38 (32%)	32; 21; 32%
3)Non-urgent symptomatic:	31 (26%)	39; 32; 10%
4)Non-urgent asymptomatic:	21 (18%)	57; 10; 10%
ERCP *n* = 42; age 71 ± 15 years; 23 females (55%); 42 in-patients (100%); 38 available rtPCR SARS-CoV-2 (91%)		
Indication		Outcome [normal; endoscopic therapy]
1)Emergency/essential:	29 (69%)	210; 97%
2)Non-urgent asymptomatic:	13 (31%)	18; 92%

Indications for endoscopies were classified on the basis of the list released by the British Society of Gastroenterology

*EGD* esophagogastroduodenoscopy, *ERCP* endoscopic retrograde cholangiopancreatography

Performed interventions were categorized according to guidance published by the British Society of Gastroenterology (non-urgent procedures were subdivided into symptomatic and asymptomatic patients) [https://www.bsg.org.uk/covid-19-advice/endoscopy-activity-and-covid-19-bsg-and-jag-guidance/]. Overall, 200 procedures (56.6%) were classified as emergent, essential or urgent. Among emergent, essential or urgent upper endoscopies, 39.6% were therapeutic and 58.4% altered management (2.4 and 4.8%, respectively, for non-urgent procedures). For lower endoscopies, 27.3% of essential/emergent and urgent procedures were therapeutic and 50% altered management (23.1 and 9.6%, respectively, for non-urgent procedures). Five malignant tumours (frequency 1.4%) were diagnosed by upper (essential/urgent: 3; non-urgent: 2) or lower endoscopy (essential/urgent: 5; non-urgent: 0). Twelve non-urgent ERCPs were done to exchange biliary stents after maximal extension of exchange intervals, and all but two ERC(P)s were therapeutic.

The results of SARS-CoV-2 rtPCR were available prior to endoscopy in 313 procedures (88.6%). After implementation of structured testing, only eight from 302 (2.6%) non-emergency procedures were performed without known COVID-19 status. No patient scheduled for endoscopy tested positive for SARS-CoV-2 either before or after endoscopy. There occurred no infection of any endoscopy team member during the described period. Table 2 presents endoscopies performed in patients with and without rtPCR test results. A significantly higher proportion of patients without test results were investigated within 6 h of presentation (*P* < 0.001). However, there was no higher probability for a clinically significant finding or need for endoscopic haemostasis for emergency patients without test results (both *P* > 0.05). When we compared endoscopic procedures with a historic cohort (31 days from 23 March to 22 April 2019), we did not notice any relevant differences between both cohorts (Table 2).

**Table 2:** Comparison of endoscopic procedures in patients with or without known COVID-19 status

	rtPCR result available	rtPCR result not available	Historic cohort 2019
Patients (*n*)	313 (89%)	39 (11%)	368 (100%)
Gender (m/f)	179 (57%)/134 (43%)	26 (67%)/13 (33%)	227 (62%)/141 (38%)
Age	62 ± 18 (1–95)	64 ± 15 (30–91)	63 ± 16 (3–88)
In-patient / out-patient	231 (74%)/82 (26%)	25 (64%)/14 (36%)	262 (71%)/106 (29%)
*Timing of endoscopy*			
< 6 h of symptom onset	26 (8%)	14 (36%)	29 (8%)
6–24 h of symptom onset	42 (13%)	3 (8%)	37 (10%)
Routine	245 (78%)	22 (56%)	302 (82%)
*Endoscopic procedure*			
EGD	161 (51%)	23 (59%)	191 (52%)
Colonoscopy	106 (34%)	12 (31%)	134 (36%)
ERCP	38 (12%)	4 (10%)	30 (8%)
EUS	8 (3%)	0 (0%)	13 (4%)
*Outcome emergency procedure*	N = 68	N = 16	N = 66
Clinically significant finding	56 (82%)	12 (75%)	43 (65%)
Endoscopic haemostasis	22 (32%)	7 (44%)	21 (32%)
Biliary/pancreatic intervention	13 (19%)	3 (19%)	4 (6%)
Other endoscopic therapy	3 (4%)	1 (6%)	8 (12%)
*Outcome routine procedures*	N = 245	N = 23	N = 302
Clinically significant finding	60 (25%)	4 (17%)	78 (26%)
Endoscopic haemostasis	3 (1%)	0 (0%)	1 (0.5%)
Biliary/pancreatic intervention	28 (11%)	1 (4%)	20 (8%)
PEG/feeding tube	8 (3%)	2 (9%)	5 (2%)
Polypectomy	18 (7%)	2 (9%)	43 (14%)
Other endoscopic therapy	7 (3%)	0 (0%)	9 (3%)

In addition to results from the first wave of COVID-19 in Germany, results of a historic cohort (pre-COVID-19 era) are presented. The number of endoscopies performed in this 31-day period was comparable with the number of procedures in the 49-day period 1 year later

Clinically significant findings: life-threatening conditions; result that changes patient management; gastrointestinal ulcers; cancer or polyps ≥ 10 mm; reflux esophagitis ≥ Los Angeles C

*EGD* esophagogastroduodenoscopy, *ERCP* endoscopic retrograde cholangiopancreatography, *EUS* endoscopic ultrasound; *PEG* percutaneous endoscopic gastrostomy

### The year 2021

**Fig. 1:**
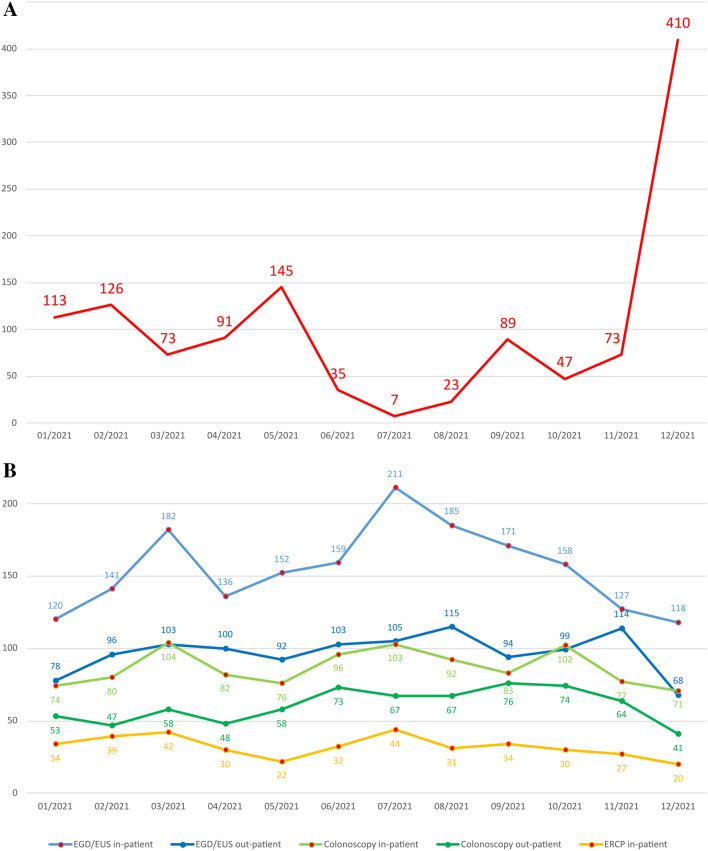
Endoscopies performed in 2021. The 7-day incidence rate for the first day of every month is shown in **A**. Number of endoscopies carried out by month and type of procedure are shown in **B**. *EGD* esophagogastroduodenoscopy, *ERCP* endoscopic retrograde cholangiopancreatography, *EUS* endoscopic ultrasound

In the year 2021, including the complete wave three and significant parts of waves two and four of the pandemic in Germany (35 of 52 weeks with a peak 7-day incidence rate of 440.9 in Saarland), more than 5100 endoscopic procedures were performed (Fig. 1). According to our testing strategy, a COVID-19 test result was available for all patients prior to endoscopy. Patients broadly accepted pre-endoscopy testing even during the low-incidence period in the summer and rising percentage of vaccinated patients. Interestingly, in this period only two asymptomatic out-patients had a positive PCR test result (a total of 1893 out-patient procedures were performed during this period). No endoscopy staff members were infected. Of note, one of the asymptomatic emergency patients (swallowed battery) with negative rapid antigen test was positive in a simultaneously performed rtPCR test (result available the next day).

## Discussion

The reduction of endoscopies performed during COVID-19 pandemic is thought to (i) save resources needed for COVID-19 patients and (ii) minimize the risk of infection for patients and endoscopy team members. However, lock-down of endoscopy units during pandemic might confront us with difficult to manage numbers of patients afterwards. This strategy also implies that the pandemic of COVID-19 will soon be over, and this assumption might not necessarily be correct. Thus, we are in need of simple and reliable screening tools that will allow functioning of endoscopy units without posing risks to patients or to the endoscopy teams. Overall, gastrointestinal endoscopy appears to be relatively safe when adequate protective measures are used [5, 6], but these measures significantly alter the workflow and potentially impair the quality of endoscopies.

Here we show that a moderate or even no reduction of the endoscopy volume based on the structured rtPCR-based SARS-CoV-2 testing on nasopharyngeal swabs is feasible. However, the retrospective design of the study, and the lack of a structured follow-up for COVID-19 infection after the endoscopic procedure limit the validity of the study. The reduction of endoscopy volume by 43% during the first wave is substantially lower than the reported reductions of about 80% [2]. In 2020 the American Gastroenterological Association (AGA) has released recommendations on the use of pre-procedure SARS-CoV-2 testing and suggested implementation of a pre-testing strategy in regions with intermediate prevalence (0.5–2%) of asymptomatic infections [7]. In the recently published updated version of the ESGE (European Society of Gastrointestinal Endoscopy) position statement on gastrointestinal endoscopy and COVID-19 a negative viral test (PCR or isothermal nucleic acid amplification; always in symptomatic patients) or documentation of full COVID-19 vaccination / recovery from infection within 6 months (not in symptomatic patients) is advised prior to gastrointestinal endoscopy [8]. Testing strategies based on rapid antigen test are not recommended [8].

However, access to the PCR tests, timely result delivery and costs might be potential limitations in real-life. Whereas our universal testing strategy reduces the probability of a missing test and captures patients with asymptomatic breakthrough infection, limited testing capacities in some areas, cost aspects, and low frequency of positive tests among asymptomatic person argue against universal testing. Given the low frequency of pre-endoscopy positive tests, it can be speculated that FFP-2 masks regularly used by the endoscopy team members during endoscopy and by patients after the procedure might be enough to prohibit spreading of the virus.

During the initial period of COVID-19, most endoscopies without a test result were performed in the evening or on weekends when results of routine tests were not available until the next morning. Notwithstanding that rapid antigen tests are usually not recommended as pre-endoscopy test due to their low sensitivity [8, 9], we performed such tests in all patients with indication for an emergent endoscopy if the rtPCR test result was not available. Based on our data and recent publications on the timing of endoscopy for upper gastrointestinal bleeding [10], the majority of endoscopies (except for suspected variceal haemorrhage with haemodynamic instability and severe septic shock due to cholangitis) can be safely deferred until the rtPCR result is available. Since false-negative rtPCR results are possible [11], we additionally recommend the use of standardized questionnaires to assess patients for COVID-19 symptoms and to adapt PPE.

## Conclusions

A structured pre-endoscopy SARS-CoV-2 testing strategy is feasible in the clinical routine of an endoscopy unit. This strategy safely allowed almost unrestricted continuation of endoscopic procedures even in the presence of high incidence rates. However, given the low frequency of positive tests, the absolute effect of pre-endoscopy testing on viral transmission may be low when FFP-2 masks are regularly used.

## Data Availability

The datasets used and analysed during the current study are available from the corresponding author on reasonable request.

## Abbreviations

rtPCR: Reverse transcriptase polymerase chain reaction
ERCP: Endoscopic retrograde cholangiopancreatography
EUS: Endoscopic ultrasound
PEG: Percutaneous endoscopic gastrostomy
PPE: Personal protective equipment
